# How do positive and negative emotions influence children’s and adolescents’ arithmetic performance?

**DOI:** 10.1371/journal.pone.0309573

**Published:** 2025-04-17

**Authors:** Nurit Viesel-Nordmeyer, Patrick Lemaire

**Affiliations:** Aix-Marseille University & CNRS, Marseille, France; Universite du Quebec a Montreal, CANADA

## Abstract

This study is the first to investigate how negative and positive emotional states influence children’s arithmetic performance and age-related differences therein. Children aged 8–14 (*n* = 149) were asked to verify true/false, one-digit addition problems (i.e., 8 + 2 = 10. True? False?) which were superimposed on emotionally negative, positive, or neutral pictures. The main results showed that (a) both positive and negative emotions impaired children’s arithmetic performance, (b) deleterious effects of negative emotions were larger than those of positive emotions, (c) effects of emotions were modulated by the type of (true/false) problems, (d) effects of emotions on current trials were influenced by emotions on immediately preceding trials, and (e) effects of emotions as well as their trial-to-trial modulations changed with children’s age. These findings have important implications for further our understanding of effects of emotions in children’s arithmetic and how these effects change as children grow older.

## Introduction

The present study examined how emotional states influence arithmetic performance in eight-to-14-year-old children. In the last decades, many studies investigated the influence of emotions on cognitive performance in adults [1,2 for reviews] as well as in children and adolescents [3,4]. In a wide variety of cognitive domains such as attention, memory, reasoning, decision making, previous studies found that emotions can increase or decrease both adults’ and children’s performance. Surprisingly, very few studies examined the role of emotions – especially emotional states − in arithmetic and even fewer studies investigated age-related changes in this role during childhood and adolescence. Most important, no previous study examined the role of both positive and negative emotional states on performance while children and adolescents of different ages are solving arithmetic problems. Thus, we ignore whether positive and negative emotional states influence children’s arithmetic performance to the same extent and how this influence changes with age. This is surprising given the importance of arithmetic in both adults’ and children’s daily life and at school. The present study aimed at filling this gap by documenting how negative and positive emotional states influence children’s arithmetic performance and how this influence changes in participants aged eight to 14 years old. Before outlining the logic of the present study, we review previous findings on the effects of emotions first on cognitive performance and age-related changes in these effects and second in the specific domain of arithmetic.

### Age-related changes in effects of emotions on cognitive performance

Emotions can be defined as “*episodic, relatively short-term biologically based pattern of perception, experience, physiology, action, and communication that occur in response to specifically physical and social challenges and opportunities*” [5, p. 468]. These patterns of perception and experience are subjectively valenced as pleasant (positive) or unpleasant (negative). Numerous studies found that emotions influence participants’ performance in a wide variety of cognitive domains, including attention, memory, reasoning, decision making, both in adults [1,2,] for overviews] and in children and adolescents [3]. In children and adolescents, like in adults, emotions can enhance, deteriorate, or have no effects on performance in different cognitive domains [3,6–11]. For example, Vasa et al. [11] found that 12–17-year-old adolescents recalled better positively and negatively valenced pictures than emotionally neutral pictures. This was also found in four-year-old children [10], in five to six-year-old children [12], and in eight- to 12-year-old children [8,13]. As another example, Howe et al. [6] found poorer recall performance in seven- and 11-year-old children for emotionally negative words relative to neutral words, and Leventon et al. [13] found no effects of emotions on recall performance in five- to eight-year-old children.

Effects of emotions in both children and adults are usually accounted for by assuming that emotions capture participants’ attention [8,14,15]. This may enhance crucial cognitive mechanisms used to accomplish the target cognitive task when emotions are task relevant (e.g., categorization of emotional and non-emotional words into “emotionally positive or negative words”). However, when emotions are task irrelevant (e.g., emotional images which are presented prior to the target task and are completely unrelated to the target cognitive task), they grab participants’ exogeneous attention and interrupt/slow down cognitive mechanisms involved in the target task, and/or would generate a competition of resources between irrelevant emotional processing and relevant cognitive processing. As a result, participants are impaired in accomplishing the target task [16–18].

Besides attention capture, effects of emotions on cognitive performance can also be attributed to emotions influencing participants’ motivation to engage in executing the target task [19–21]. As suggested by Pekrun’s Control Value Theory [CVT, 4,19–21], effects of positive activating emotions (e.g., enjoyment) on participants’ performance may be mediated by participants’ engagement in learning. In contrast, negative emotions (e.g., boredom) may exert their deleterious effects via lowering participants’ engagement in learning or while accomplishing cognitive tasks.

### Age-related differences in effects of emotion on arithmetic

Two lines of evidence suggest that negative and positive emotions influence arithmetic performance, both in adults and during childhood and adolescence. First, numerous studies found correlations between different achievement-related emotions (emotional traits) and arithmetic performance [22–33]. For example, Pekrun et al. [28–30] found that a variety of positive emotions (enjoyment, hope, pride, relaxation, assurance, relief) were positively related to children’s and adolescents’ math performance whereas a variety of negative emotions (anger, anxiety, shame/guilt, boredom, hopelessness, disappointment) were negatively related with children’s and adolescents’ math performance. These relations were reciprocal and were found to be stable in longitudinal studies over a large age span in children and adolescents [28–30,34].

Also, numerous studies found negative correlations between math anxiety (i.e., feelings of tension specifically associated with math) and math performance [22–24,32, for reviews]. Such correlations showed that more math anxious individuals obtain poorer math performance, but also that poor math performance is associated with the development of math anxiety. Note that these correlations hold whatever participants’ age, although they tend to increase from elementary-school children to high-school children [22,32]. Also, studies found significant correlations between individuals’ arithmetic performance and positively valenced emotional traits like math enjoyment [25,31] or math self-efficacy [26,31]. Whichever participants’ age, these correlations showed that individuals with higher math enjoyment or higher math self-efficacy perform better in math. Conversely, participants’ better math performance is also associated with increased positive math-related emotional traits.

Experimental studies provide a second line of evidence on the effects of emotions in arithmetic. In adults, these studies used standardized emotional picture systems that previous research had established to be valid to induce participants’ emotions [35,36, for reviews]. In these studies, participants showed impaired arithmetic performance under negative conditions compared to neutral conditions studies [37–50]. Whereas two of these studies found slightly improved arithmetic performance under positive emotions [41,46], other studies found that positive emotions had deleterious effects although to smaller magnitudes than negative emotions [37,44,48]. For example, Lallement and Lemaire [44] asked adult participants to verify simple arithmetic problems (e.g., 8 + 7 = 15. True? False) while problems were displayed superimposed on emotionally negative, positive, or neutral pictures. They found that participants were slowed down while verifying problems under both negative and positive emotions relative to neutral emotions, but much more so under negative than under positive emotions. These studies were limited to adult participants, so that we do not know if negative and positive emotions have deleterious effects also in children, whether these effects are of similar or of different magnitudes, and how these effects change with children’s age.

To date, only a few experimental studies have been carried out in children or adolescents to examine effects of emotional states on arithmetic performance [14,51–53]. Lemaire [14] asked children aged eight to 15 to verify simple arithmetic problems (e.g., 3 + 4 = 7 or 3 + 5 =11. True? False) that were displayed superimposed on emotionally neutral or negative pictures. Children in all age groups obtained poorer performance under negative than under neutral emotion conditions. Interestingly, these deleterious effects of negative emotions decreased as children grow older and were larger when current emotionally negative trials followed negative trials compared to after neutral trials. Unfortunately, Lemaire’s study did not include positive emotion conditions.

Bryan and Bryan [51] tried to determine how positive emotions influence children’s arithmetic performance. Eight- to 10-year-old children and 14.7- to 17.6-year-old adolescents were first asked to close their eyes and think of the happiest moment in their life or of the last time they felt very happy. After 45 seconds, they were asked to open their eyes and describe what they were thinking of. Following this, they were given 50 addition and subtraction problems and asked to solve as many problems as possible in five minutes. Both younger and older children solved more problems following positive emotion induction. However, lack of control for non-emotional factors makes it impossible to know whether the positive emotion group obtained slightly better performance than the no-treatment group because of positive emotions or because of other factors (e.g., the imagery task triggered focused attention and/or increased non-specific arousal) that enabled participants to solve more arithmetic problems.

Also, two studies used the same affective priming paradigm (i.e., children aged 7–13 solved arithmetic problems following display of positive, negative, and neutral words), but found inconsistent results. Rubinsten and Tannock [53] found that 7–13-year-old children were faster on solving arithmetic problems following emotionally positive than after emotionally neutral words and slower after emotionally negative words than after neutral words. However, with the same procedure, but with a much larger sample of children (*N*=96), Kucian et al. [52] did not replicate these math emotional priming effects.

Thus, we do not know yet how positive emotional states influence children’s performance while they are solving arithmetic problems, whether such emotional states have facilitative or deleterious effects, if positive emotions hinder performance less than negative emotions like they have during adulthood and if the influence of positive emotions on children’s and adolescents’ arithmetic performance changes with participants’ age. The present study was carried out to address these issues. The knowledge gained from our study is expected to provide helpful information for a better understanding of children’s and adolescents’ performance ability in arithmetic in different situations of their daily life.

### The present study

The present study was not pre-registered. It aimed at answering the following questions: (a) Is children’s arithmetic performance similarly influenced by positive and negative emotions? and (b) How do effects of positive and negative emotions change with children’s age? We examined 8–14-year-old participants’ performance as a function of negative, positive, and neutral emotions while they verified a series of simple arithmetic problems (e.g., 8 + 9 = 17. True? False). We used a within-trial emotion induction procedure (i.e., displaying arithmetic problems superimposed on pictures) as this procedure proved successful and yielded valid and sensitive measures of effects of emotions on performance in past research both in arithmetic [14,37,43,44] as well as outside arithmetic [35].

We tested two hypotheses. First, negative emotions were expected to impair children’s execution of arithmetic problem-solving processes and to lead to decreased arithmetic performance, as previously found for children and adolescents in Lemaire [14] and in several adult studies [39–44,46,48]. Concerning effects of positive emotions, given the impossibility to draw clear conclusions from the very few previous studies in children and adolescents [51–53] or from previous adult studies [37,41,44,46,48], we cannot hypothesize that positive emotions will enhance or impair children’s and adolescents’ arithmetic problem-solving processes. Results were expected to tell whether positive emotions facilitate children’s performance or impaired their arithmetic performance. Specifically, if children’s positive emotions lead them to focus more efficiently on the target arithmetic task and to execute arithmetic problem-solving processes more efficiently, we should find beneficial effects on arithmetic performance. Alternatively, if positive emotions grab children’s attention and distract them away from the target arithmetic task, like negative emotions, and impair their performance, as was already found in adults [37,43,44,48], we expected deleterious effects of positive emotions. We also tested the possibility that positive emotions have no effects on children’s arithmetic performance, replicating previous findings [52]. In sum, the present data were collected to determine whether both negative and positive emotions influence children’s arithmetic performance, to what extent (or how much?) they do so, and in which directions these influences go (i.e., deleterious vs. enhancement effects).

The second hypothesis under test here concerns developmental changes. We hypothesized that effects of negative emotions on arithmetic performance would decrease as children grow older. Not only this would replicate previously found smaller effects of negative emotions in older children [14], but also from age-related cognitive growth (i.e., increased arithmetic fluency, better emotion regulation, more efficient executive control) during childhood. Given no previous findings on age-related changes in effects of positive emotions in arithmetic, we made no specific developmental predictions regarding age-related changes in effects of positive emotions. The data were expected to help us determine whether these effects increase, decrease, or remain stable as children grow older.

Of additional interest here were the sequential modulations of effects of emotions and age-related changes in these modulations. Recall that Lemaire [14] found that effects of negative emotions on children’s arithmetic performance were larger on current trials following negative emotion trials relative to after neutral emotion trials. We wanted to determine if such sequential modulations of effects of negative emotions are replicated, so as to know whether these effects generalize to the contexts where children experience negative, positive, and neutral emotions, and how these sequential modulations might differ for positive and negative emotions. Interestingly, such sequential effects inform how children approach each trial. That is, children can either prepare themselves in-between trials for the incoming trial or process each trial independently of the preceding trial. When they prepare themselves for the next trial following an emotional trial, effects of negative emotions decrease. When they approach each successive trial independently, emotional effects on current trials remain unchanged whichever the emotional valence of the immediately preceding trials. The present experiment enabled us to test these two possibilities for current positive and negative emotions and how this might change with children’s age.

## Method

### Participants

We tested 149 participants from four different grades in public schools in the South of France (grades 3, 5, 7, and 9). Children were of four different age groups: 8-year-olds, 10-year-olds, 12-year-olds, 14-year-olds (see participants’ characteristics in Table 1). The sample size was determined following our previous study on emotion and arithmetic in children (14), where effect size of emotion on arithmetic performance was *η*²*p* = 0.356. With one between-participants factor (age group) and two within-participants factors (problem type and emotion), our design could achieve 95% power with 56 participants. In order to exceed this criterion, we recruited 149 participants. Written informed consents were obtained from participants’ parents, and oral consents were obtained from each participant. This research was approved by the National and Local Ethics Committees (Ref #: *Comité Nationale pour la Recherche Impliquant des Personnes Humaines,* CNRIPH 2022-04-14-014) and was conducted in accordance of the Declaration of Helsinki. The testing was conducted between the 15th September 2022 and the 10th February 2023.

**Table 1:** Participants’ characteristics.

Characteristics	8 y.o. (*N* = 35)	10 y.o. (*N* = 40)	12 y.o. (*N* = 34)	14 y.o. (*N* = 40)	*F*
*N* (females)	35 (19)	40 (16)	34 (19)	40 (21)	--
Mean age in years.months (*SD*)	8.3 (0.30)	10.5 (0.42)	12.4 (0.35)	14.2 (0.41)	1696.160^**^
Age Range	7.92–8.92	9.58–11.42	11.92–13.75	12.58–15.08	--
Grade	Third	Fifth	Seventh	Ninth	--
TTR Arithmetic Fluency (*SD*)	46 (15.09)	84 (21.05)	97 (21.05)	109 (22.19)	65.821^**^
Math stimuli RTs	7403 (5741)	5056 (3767)	4249 (2770)	2935 (1583)	.457^***^

*Note*. TTR = Arithmetic Tempo Test [54];

**p <.001.

### Stimuli

*Arithmetic problems* Each child solved 96 problems. The basic set of problems was the same as the one used by Lemaire [14]. It included 12 individual addition problems presented in a standard form (i.e., *a* + *b*) with the operands *a* and *b* being one-digit numbers (e.g., 3 + 4). Each individual problem was presented in *a* + *b* and *b* + *a* version (e.g., 3 + 4 and 4 + 3). Each problem was presented with its correct sum (e.g., 3 + 8 = 11) and with an incorrect sum (e.g., 3 + 8 = 13). Proposed sums in false problems had a deviation of ± 1 or ± 2 units from correct sums. Thus, the 12 individual problems were presented in two versions (*a* + *b*, *b* + *a*) and with a true and a false answer, yielding 48 problems. Participants solved these 48 problems in neutral emotion condition (*N* = 48) as well as half in negative (*N* = 24) and half in positive (*N* = 24) emotion conditions. Thus, each participant solved 96 addition problems. Following previous works in arithmetic [54–57] problems were selected so that no tie problems (e.g., 3 + 3 = 6) were used; none of the operands were equal to 0, 1, 2, or 5; and none of the false answers were table-related products (e.g., 3 + 4 = 9). An additional set of 10 practice problems (similar to but different from experimental problems) was selected.

*Pictures*. One hundred and six pictures (96 for experimental trials, 10 for practice trials) for the experimental phase and 53 additional (48 for experimental trials, five for practice trials) for the picture-judgment task were selected from the Developmental Affective Photo System [DAPS; 57]. We used the *Developmental Affective Photo System* [DAPS; 57], one of the Standardized Emotion Elicitation Databases, devised after the *International Affective Picture System* [IAPS; 58] developed for adults. This type of emotion elicitation databases has been validated numerous times [35,36, for reviews] with both behavioral (ratings of valence and arousal; eye tracking; facial expressions) and physiological measures (including heart rate variability, skin conductance level, electromyography, EEG, respiratory-related evoked potentials, and fMRI) in numerous studies. The DAPS pictures that we selected displayed different types of negative (e.g., anger, sadness) or positive (e.g., happiness, excitement) emotions. No distinction was made here between emotions as this study was the first to compare effects of negative and positive emotional states while children are solving arithmetic problems. Half the pictures were emotional and half were neutral. Half the emotional pictures were emotionally negative (*mean* valence = 4.4; *SD* = 0.17 and *mean* arousal = 2.0; *SD* = 0.14), and half positive (*mean* valence = 1.7; *SD* = 0.23 and *mean* arousal = 2.0; *SD* = 0.27). The emotionally neutral pictures had a *mean* valence of 2.7 (*SD* = 0.30) and *mean* arousal of 3.75 (*SD* = 0.33). Mean arousal for positive and negative pictures were not different, both overall (*F*=.001; *p* =.97), and for each true (*F*= 1.04; *p* =.32), and false problems (*F*=.77; *p* =.39,), separately. Half the true and false problems were presented with emotionally neutral pictures, a quarter with emotionally positive pictures, and a quarter with emotionally positive pictures. Images presented with true and false problems were matched on mean valence and arousal (see Table 2).

**Table 2:** Mean valence and arousal of emotionally neutral, negative, and positive conditions for true and false problems (*SDs* in parentheses).

Emotion Condition	True problems	False problems	*Fs*
*Valence*			
Neutral	2.64 (0.25)	2.6 (0.23)	5.07 ns
Negative	4.42 (0.23)	4.41 (0.21)	0.035 ns
Positive	1.74 (0.25)	1.74 (0.22)	0.001 ns
*Arousal*			
Neutral	3.76 (0.35)	3.75 (0.31)	0.006 ns
Negative	2.00 (0.14)	2.01 (0.14)	0.017 ns
Positive	2.08 (0.22)	1.93 (0.29)	1.98 ns

Note. In DAPS, valence ranges from 1 (very happy) to 5 (very unhappy), and arousal ranges from 1 (very excited) to 5 (very calm).

*Arithmetic fluency* The Tempo Test Rekenen [TTR; 59] was used to evaluate participants’ arithmetic fluency. Children had to solve a maximum of four series of arithmetic operations (addition, subtraction, multiplication, division) each including up to 40 problems. For every series, children were given one minute to solve as many of the 40 problems as possible.

### Procedure

**Fig 1:**
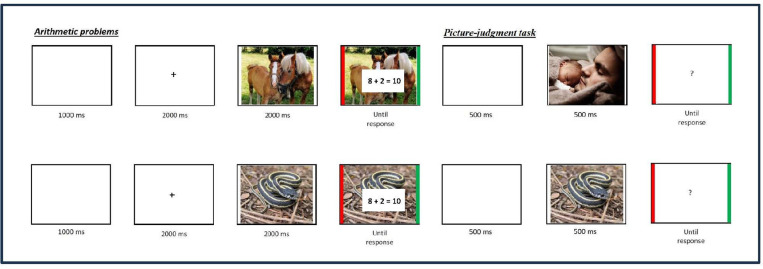
Illustration of procedure (images are from the OASIS database and were not used in this experiment as images used in this experiment were from DAPS).

The main experiment was run on a Windows 10 Microsoft Surface Go2 Touch-Screen (10.5 inches), intel® Core™, M3-8100Y. Children were tested in groups of ten to 20 participants in their classroom at school. Participants were told that they will see pleasant and unpleasant pictures and will complete an arithmetic problem-verification task. Each trial started with a 1000-ms blank screen and a 2000-ms fixation cross in the center of the screen (see illustration of a trial in Fig 1). A picture was then displayed for 2000-ms. Then, addition problems together with a proposed sum appeared superimposed on emotionally neutral (e.g., landscape), positive (e.g., kittens), or negative (e.g., a car accident) pictures until the participant’s response. Participants had to indicate if the proposed result was correct or not as quickly and as accurately as possible. To do this, they had to press a delimited area (a 1.4-cm thick green stripe) on the right side of the Touch Screen if the proposed result was correct and a delimited area (a 1.4-cm thick red stripe) on the left side of the Touch Screen, if the proposed result was incorrect. After the participant answered, a blank screen was displayed for 500 ms, a picture with the same (neutral, positive, or negative) emotional valence was displayed for 500 ms together with a question mark. Participants had to indicate if the picture was the same or different than the picture presented before and during the arithmetic task (half the pictures were the same, and half were different). Participants started with a practice session in which they verified 10 similar (but not the same) equations (five true, five false, four presented with a neutral picture, three with a negative picture, and three with a positive picture). Then, participants completed 96 trials, divided into two blocks of 48 trials each (half neutral and half emotional, randomly presented). Participants took a short break in-between the two blocks. The problem and pictures remained displayed on the screen until participants’ response. All problems were randomly presented to each participant.

At the end of the experiment, each participant saw 18 emotionally positive pictures (e.g., a smiling baby) in order to end the experiment in a positive mood. Each picture included a green, a blue, or a red butterfly. On each picture, participants had to determine the colour of the butterfly. When they were ready to provide their answer, they clicked on the screen. Three coloured circles immediately appeared on the screen, and participants were asked to press the green circle (right side of the screen) if the butterfly on the picture was green, on the blue circle (middle of the screen) if the butterfly on the picture was blue, or on the red circle (left side of the screen) if the butterfly was green.

After this problem-verification task, participants’ arithmetic fluency was assessed with the *Arithmetic Tempo Test* [59]. This test includes four sets of 40 arithmetic problems each, with each set made of only addition, subtraction, multiplication, or division problems. For each set, children had to provide the correct answer to as many problems as possible in 1-min. Finally, they had one minute to solve as many problems as possible in a mixed set of addition, subtraction, multiplication, and division problems. The sum of correctly solved problems was calculated for each individual.

## Results

Data analyses aimed at addressing the following issues: (a) Is children’s arithmetic performance influenced by emotions and, if it does, do negative and positive emotions have the same impact? (b) Are effects of emotions on current trials sequentially modulated by emotions on immediately preceding trials? and (c) Do effects of emotions and their sequential modulations change as children grow older?

### Age-related differences in effects of emotions on arithmetic performance

Generalized linear mixed-effect models were conducted using the lme4 [60] and lmerTest [61] packages in *R* [Version 4.2.2, 62]. Participants’ mean response times of correctly solved problems and percentages of errors for true and false problems under emotionally neutral, negative, or positive condition in each age group are shown in Table 3.

**Table 3:** Mean solution times and percentages of errors for true and false problems under (neutral, negative, and positive) emotion conditions as a function of children’s age while solving true and false problems.

Age x Problem	Latencies (in ms)					% Errors				
	Neutral	Negative	Positive	Negative -Neutral	Positive -Neutral	Neutral	Negative	Positive	Negative -Neutral	Positive -Neutral
*8-year-olds*										
True	7062	8099	7520	1037	458	15.1	19.3	19.0	4.2	3.9
False	7762	9115	8052	1353	290	19.4	17.4	15.2	-2.0	-4.2
*Means*	7412	8607	7786	1195	374	17.35	18.4	17.1	2.2	0.2
*10-year-olds*										
True	4661	5263	4833	602	172	10.0	10.2	10.4	0.2	0.4
False	5455	8074	5280	2619	-175	10.6	9.0	10.0	-1.6	-0.6
*Means*	5058	6669	5057	1611	-1.5	10.3	9.6	10.2	-0.7	-0.1
*12-year-olds*										
True	4005	4677	4626	672	621	7.6	5.4	6.4	-2.2	-1.2
False	4493	5631	5084	1138	591	7.6	8.1	8.9	0.5	1.3
*Means*	4249	5154	4855	905	606	7.6	6.8	7.7	-0.9	0.1
*14-year-olds*										
True	2726	3052	2875	326	149	4.9	2.5	6.5	-2.4	1.6
False	2935	3505	3094	570	159	5.8	3.5	3.1	-2.3	-2.7
*Means*	2830	3279	2985	448	154	5.4	3.0	4.8	-2.4	-0.6

To examine effects of emotions on children’s performance, both response times (in ms) and correct responses (0 = error, 1 = accurate) were modelled with emotions of the current trials (neutral = 0, negative = 1, positive = 2) as main predictors. Age as well as problem veracity (0 = false, 1 = true) were also included. Further, interactions of emotions with both other predictors (Age x Emotion, Emotion x Veracity, Age x Emotion x Veracity) were added. Each model was computed first with the full sample (*N* = 149) and second separately for each age group (8 y.o., 10 y.o., 12 y.o., 14 y.o.). Specifically, all of these models were estimated with a classical general mixed-model approach [63] using maximum likelihood (MLM). In each of the generalized linear mixed-effect model, only participants were set as random slope to preserve model parsimony. Specifically, a beneficial effect of setting more variables on slope could be rejected based on model fit comparison and likelihood ratio tests including AIC and BIC [64]. All models were also computed with multilevel Bayesian statistic approach [65] (S1-S10 Tables), including No-U-Turn [NUTS; 66]. No differences in the results were found. Further, all analyses were re-run with arithmetic fluency scores as covariates. Analyses showed similar patterns for effects of emotions on performance and for the Age x Emotion interaction.

#### Latencies.

Participants were faster as they grow older (*β* = -715.78; *SE* = 81.57; *t* = -8.78; *p* <.001) and were faster on true than on false problems (*β* = -686.03; *SE* = 148.60; *t* = -4.62; *p* <.001). More in*t*erestingly, the main effect of emotions (*β* = 682.06; *SE* = 221.87; *t* = 3.07; *p* =.002) was significant.

*Effects of negative emotions* Overall, negative emotions led children to be slower, especially while solving false problems, and these deleterious effects of negative emotions decreased with children’s increasing age. More specifically, children were 1040 ms slower under negative than under neutral emotions (*β* = 3812.20; *SE* = 577.04; *t* = 6.61; *p* <.001). Also, the Age x Emotion interaction was significant (*β* = -40.59; *SE* = 18.70; *t* = -2.17; *p* =.03). Dele*t*erious effects of negative emotions decreased as children grow older. Thus, relative to neutral emotion conditions, 8-year-olds were 1195 ms slower under negative emotion condition (*β* = 1400.84; *SE* = 349.43; *t* = 4.01; *p* <.001), 10-year-olds were 1611 ms slower (*β* = 2594.12; *SE* = 232.15; *t* = 11.18; *p* <.001), 12-year-olds were 905 ms slower (*β* = 1105.80; *SE* = 182.34; *t* = 6.06; *p* <.001), and 14-year-olds were 448 ms slower (*β* = 566.38; *SE* = 84.93; *t* = 6.67; *p* <.001).

Contrast analyses showed that effects of negative emotions differed between eight- and 10-year-olds (*β* = 1193.05; *SE* = 315.7; *z* = 3.78; *p* <.001), eight- and 14-year-olds (*β* = -834.34; *SE* = 311.86; *z* = -2.68; *p* =.01), 10- and 12-year-olds (*β* = 1487.9; *SE* = 308.5; *z* = 4.82; *p* <.001), and 10- and 14-year-olds *(β* = 2027.4; *SE* = 292.7; *z* = 6.93; *p* <.001). The deleterious effects of negative emotions were not different between eight-year-olds and 12-year-olds (*β* = -294.84; *SE* = 326.7; *z* = -.9; *p* =.37) and were marginally significant between 12-year-olds and 14-year-olds *(β* = 539.5; *SE* = 304.5; *z* = 1.77; *p* =.08). Thus, following increased deleterious effects of negative emotions between eight- and 10-year-olds, deleterious effects decreased from 10- to 12-year-olds and from 12-year-olds to 14-year-olds, though to somewhat lower extents after age 12.

Effects of negative emotions on children’s latencies were modulated by the *type of problems*, as seen in significant Problem Veracity x Emotion interactions both in the full sample (*β* = -571.48; *SE* = 186.67; *t* = -3.06; *p* <.001) and in each age group. Indeed, effects of negative emotions were larger for false problems than for true problems in eight- (1353 ms vs. 1037 ms; *β* = -1133.30; *SE* = 404.34; *t* = -2.80; *p* =.01), 10- (2619 ms vs. 602 ms; *β* = -2781.36; *SE* = 268.14; *t* = -10.37; *p* <.001), 12- (1138 ms vs. 672 ms; *β* = -926.28; *SE* = 209.41; *t* = -4.42; *p* <.001) and 14-year-olds (570 ms vs. 326 ms; *β* = -449.91; *SE* = 97.41; *t* = -4.62; *p* <.001).

*Effects of positive emotions* Overall, positive emotions impaired 12 and 14 year-olds but not younger participants, and these deleterious effects of positive emotions interacted with whether participants verified true or false problems. Specifically, the main effect of positive emotions (*β* = 89.97; *SE* = 575.06; *t* =.16; *p* =.88) and the Positive Emotion x Age interaction (*β* = -550.46; *SE* = 816.31; *t* =.67; *p* =.50) on children’s latencies did not come out significant in the analyses of the full sample (*β* = 89.97; *SE* = 575.06; *t* =.16; *p* =.88). In general, children were equally fast under positive and neutral emotion conditions. However, separate analyses in age group showed small deleterious effects of positive emotions in 12-year-olds and in 14-year-olds. More specifically, relative to neutral emotions, 12-year-olds were 606-ms slower under positive emotions (*β* = 579.63; *SE* = 183.01; *t* = 3.17; *p* =.002), and these emotion effects were larger for true than for false problems (621 ms vs. 591 ms; *β* = -467.95; *SE* = 210.56; *t* = -2.22; *p* =.03). Also, 14-year-olds tended to slow down under positive emotions (*β* = 147.42; *SE* = 84.80; *t* = 1.74; *p* =.08), but these emotion effects were larger for false than true problems (159 ms vs. 149 ms; *β* = -208.81; *SE* = 98.33; *t* = -2.12; *p* =.03).

#### Accuracy.

Overall, participants were more accurate as they grow older (*β* =.02; *SE* =.01; *t* = 3.64; *p* <.001) and more accurate on true than on false problems (*β* =.03; *SE* =.01; *t* = 2.73; *p* =.01). However, par*t*icipants were more accurate on false than on true problems under negative emotions (*β* = -.14; *SE* =.06; *t* = 2.56; *p* =.01) compared *t*o neutral conditions. Interestingly, the main effect of emotions (*β* =.00; *SE* =.02; *t* =.30; *p* =.76) did no*t* come out significant.

Separate analyses in each age group showed that 14-year-olds were slightly more accurate (+2.4%) in the negative than in the neutral emotion condition (*β* =.02; *SE* =.01; *t* = 2.00; *p* =.05). Moreover, 14-year-olds were slightly more accurate (+0.6%) under positive than under neutral emotion condition (*β* =.03; *SE* =.01; *t* = 2.36; *p* =.02), and these emotion effects were larger for false than for true problems (*β* = -.03; *SE* =.01; *t* = -2.51; *p* =.01). Also, eight-year-olds were slightly more accurate under positive than under neutral emotions (0.2%, *β* =.04; *SE* =.02; *t* = 2.08; *p* =.04).

### Age-related differences in sequential modulations of effects of negative and positive emotions

**Fig 2:**
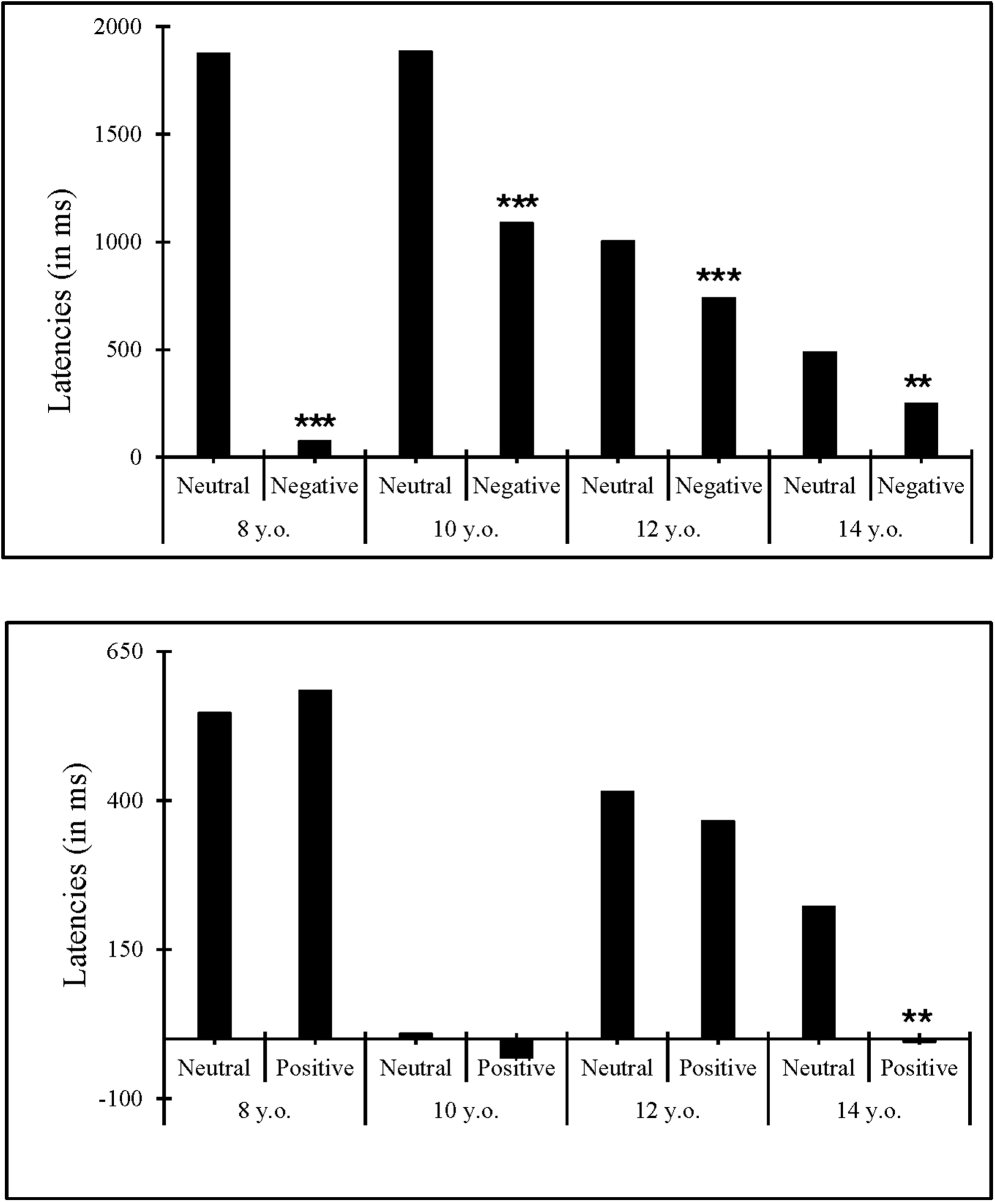
Sequential modulation of effects of emotions on current problems in each group. Effects of (a) negative (i.e., negative – neutral) and (b) positive (i.e., positive – negative) emotions on current trials as function of emotional valence on immediately preceding trials. *Note*: *** *p* ≤.001; ** *p* ≤.01.

The goal of this series of analyses was to determine whether the effects of emotions found on latencies on current trials were modulated by the type of immediately preceding trials (neutral vs. emotion), as well to determine if such sequential modulations differ for positive and negative emotions, and/or with participants’ age (see Fig 2).

Latencies larger than participants’ mean (+ 2.5 *SD;* 1.4*%*) were excluded. Sequential modulations of effects of negative emotions and of positive emotions were analyzed separately, with the following mixed-design ANOVAs, involving 4 (Age Group: eight-year-olds., 10-year-olds, 12-year-olds, 14-year-olds) × 2 (Previous Trial: Neutral, Emotion) x 3 (Current Trial: Neutral, Emotion), with Age Group as the only between-participants factor.

 Regarding sequential modulations of effects of negative emotions, the crucial Age Group x Previous Emotion x Current Emotion interaction came out significant (*F*(3, 145) = 3.37, *p* =.02, $\eta^{2}$=.07). Breakdown analyses in each age group revealed effects of negative emotions (i.e., negative – neutral) on current trials went from 1875 ms after neutral trials (*SE* = 509.29, *t* = -3.68, *p* <.001) down to 75 ms after negative emotion trials (*SE* = 509.29, *t* = -.15, *p* =.89) in eigh*t*-year-olds. Moreover, effects of negative emotions on current trials went from 1884 ms after neutral trials (*SE* = 325.62, *t* = 5.79, *p* <.001) down *t*o 1088 ms after negative emotion trials (*SE* = 325.62, *t* = 3.34, *p* <.001) in 10-year-olds, and from 488 ms (*SE* = 94.13, *t* = 5.18, *p* <.001) af*t*er neutral *t*rials down to 248 ms after negative emotion trials (*SE* = 94.13, *t* = 2.64, *p* =.01) in 14-years-olds. The sequential modulations of effects of nega*t*ive emotions did not come out significant in 12-year-olds (Previous Trial x Current Trial: *F*(1, 33) =.84, *p* =.37, $\eta^{2}$=.03), although they went in the same directions as in the other age groups, decreasing from 1004 ms after neutral trials (*SE* = 237.03, *t* = 4.24, *p* <.001) down to 740 ms after negative emo*t*ions trials (*SE* = 237.03, *t* = 3.12, *p* =.01).

Regarding sequential modulations of effects of positive emotions, the Age Group x Previous Emotion x Current Emotion (*F*(3, 145) =.09, *p* =.97, $\eta^{2}$=.02) and the Previous Emotion x Current Emotion *(F*(3, 145) =.13, *p* =.72, $\eta^{2}$= 8.991×10^-4^) did not come out significant. Nevertheless, breakdown analyses in each age group showed that the Previous Emotion x Current Emotion interaction was significant in 14-year-olds (*F*(1, 39) = 4.51, *p* =.04, $\eta^{2}$=.10). Effects of positive emotions went from 222 ms after neutral trials (*SE* = 83.95, *t* = 2.64, *p* =.01) down to -5 ms after positive emotion trials (*SE* = 83.95, *t* = -.06, *p* =.95) in 14-year-olds. Although effects of positive emotions on current trials tended to change from after neutral to after positive emotion trials in each age group (i.e., from 547 ms to 584 ms in eight-year-olds, from 9ms to 32 ms in 10-year-olds, from 415 ms to 366 ms in 12-year-olds), the Previous Emotion x Current Emotion interactions were not significant in these age groups (*Fs* <.02).

## General discussion

The present study is the first that examined the influence of both negative and positive emotions on arithmetic performance in children’s and adolescents, determined whether effects of both negative and positive emotions on current trials change as a function of emotions on immediately preceding trials, and age differences therein. Children aged eight to 14 were asked to verify true/false, one-digit addition problems (i.e., 8 + 2 = 9. True? False?) which were superimposed on emotionally negative, positive, or neutral pictures. The main findings revealed deleterious effects of both negative and positive emotions on participants’ arithmetic performance. These deleterious effects were larger for negative than for positive emotions and interacted with children’s age as well as with whether the problems to verify were true or false problems. Also, effects of both negative and positive emotions on current trials interacted with emotions on previous trials and with children’s age. These findings have important implications to further our understanding of how emotions influence children’s arithmetic performance and how effects of emotions change during childhood.

### Effects of emotions on arithmetic performance in children and adolescents

We found that children in all age groups obtained poorer performance under emotionally negative conditions than under neutral conditions. Such poorer performance under negative emotion conditions is consistent with previously reported deleterious effects of negative emotions on arithmetic performance both in children [14] and adults [37,39,40,43,44,46,48–50]. Most originally, we found that children also obtained poorer performance under emotionally positive conditions than under neutral conditions. This study is the first to provide direct evidence on the effects of positive emotions on children’s arithmetic performance.

Following previous proposals to account for how emotions influence cognitive performance [14,15], the present poorer arithmetic performance under both emotionally negative and positive conditions can be explained by an attentional capture mechanism [15,17,18]. That is, emotions captured participants’ attention and thereby interrupted and/or slowed down the cognitive mechanisms involved in the target arithmetic task. More specifically, following processing of emotional pictures, upon problem display, participants had to disengage from emotional processing before or while orienting their attention to the target arithmetic problems, encoding operands, calculating the correct sum, comparing the proposed and calculated sums, making a true/false answer, and providing their response. Participants executed some or all of these mechanisms more slowly and/or delayed their execution under emotion conditions, leading them to slow down and/or making more errors.

Most originally, this study is the first one to find impaired performance under positive emotions while children and adolescents solved simple arithmetic problems, and to find that smaller deleterious effects under positive emotions than under negative emotions. Note though that Lallement and Lemaire [44] recently found that positive emotions impaired young and older adults’ arithmetic performance. Also, like here in children and adolescents, Lallement and Lemaire [44] found that effects of positive emotions were smaller than effects of negative emotions. In other words, the present findings generalize to children previously found deleterious effects of emotions on arithmetic problem verification performance. Participants’ attention may have been captured by emotionally positive pictures, although to somewhat lower extent than by emotionally negative pictures. Given that we carefully matched arousal ratings for positive and negative pictures, valence-related differences cannot be explained by differences in arousal.

The deleterious effects of positive emotions on arithmetic performance found here do not mean that positive emotions always have deleterious effects in arithmetic or have deleterious effects on all mathematical activities. Indeed, previous studies showed that positively valanced emotional traits like math enjoyment [25,31,33] or math-self-efficacy [25,26] correlate with better math performance [28,30]. Whether beneficial effects of positive emotions on math performance can be found only with positively emotional traits or whether emotional states can sometimes lead to improved math performance while participants are solving math problems will have to be determined in future research. Such future research could use different types of emotion induction procedures (e.g., watching videos or reactivating emotionally positive autobiographical memories before solving math problems) and could test different discrete positive emotions (e.g., happiness, pride). Similarly, whether positive emotions may lead to improved performance of children and adolescents in some math activities or tasks and to impaired performance in other math activities or tasks could be investigated in future studies.

Our findings also showed larger effects on participants’ latencies of negative emotions on false problems than for true problems in all age groups. Also, when significant, effects of positive emotions on participants’ latencies were slightly smaller (in 12-year-old children) or slightly larger (in 14-year-old children) on true than on false problems. Thus, when emotions influenced participants’ latency, effects of emotions were larger on false than on true problems. As false problems like those tested here (i.e., with proposed answers close to correct answers) are known to be harder than true problems [67,68], they were more affected by negative emotions than true problems [43]. In other words, negative emotions influenced children’s arithmetic performance more strongly on harder false problems than on easier true problems. In contrast to negative emotions, effects of positive emotions on children’s latencies were not systematically larger for harder problems. For unknown reasons, they were of smaller magnitudes on false than on true problems in 12-year-old children and the reverse in 14-year-old children. Future studies may examine further whether interference effects of positive emotions may interact with problem characteristics in children’s arithmetic by testing different types of false problems, such as small- vs. large-split problems, or problems verifying/falsifying arithmetic rules, like parity or five rules [37,43,44].

The final findings of interest here regarding effects of emotions on arithmetic performance concerns sequential modulations of effects of emotions on current trials on arithmetic performance. More specifically, we found that effects of emotions on current trials were smaller following previous emotional trials than after neutral trials. The present sequential modulations of effects of emotions are analogous to sequential modulations of effects of distracting sounds on children’s arithmetic performance reported by Lemaire and Lee [69]. Lemaire and Lee [69] found that eight- and 10- year-old children obtained poorer arithmetic performance when they heard distracting sounds than in a silence condition. Such distracting effects on current arithmetic problems were smaller after sound trials than after silence trials. Following Lemaire’s and Lee’s [69] proposal, we assume that children here prepared themselves for the next trial to ignore the irrelevant negative or positive pictures and could focus their attention on the arithmetic problem-solving task. This likely helped them to shield against interference from emotional pictures during arithmetic problem solving.

### Age-related differences in effects of emotions on arithmetic during childhood

Deleterious effects of negative emotions changed with children’s age. More specifically, we found deleterious effects of negative emotions that were larger in 10-years-olds than in 8-years-old and that decreased with increasing age thereafter. Larger effects of negative emotions in 10-year-olds than in 8- and 12-year-old children were surprising and inconsistent with what we expected and with previous findings [14]. Further research is necessary to verify whether this developmental pattern of deleterious effects of negative emotions is specific to the present sample or whether there are true increased of effects of negative emotions in 10-year-olds and why. The general decreasing trend of deleterious effects of negative emotions on children’s arithmetic performance with increasing age in our study is consistent with previous findings [14]. This may be the result of different sources, like more efficient executive control mechanisms and better emotion regulation, that helped older children to be less strongly influenced by negative emotions.

More originally, we found different developmental changes in effects of positive emotions. Positive emotions had no influence on younger children’s arithmetic performance but had deleterious effects on our two older groups of 12- and 14-year-olds, especially 14-years-olds. More specifically, the deleterious effect of positive emotions in 12-years-olds was just marginally significant. One possible interpretation of these effects of positive emotions in our groups of older children is suggested by previous findings regarding distribution of attention across negative, positive, and neutral stimuli [70–72]. Indeed, previous research on the neurocognitive development of ignoring and attending emotional stimuli showed that adolescence is characterized by increased activation of the anterior insula for attending emotionally positive faces [73]. This suggests that our younger children may have devoted less attention to emotionally positive pictures than our older participants, leading the former to not be influenced by positive emotions during arithmetic problem solving. In contrast, if our older groups processed emotionally positive pictures more deeply, they may have, as a consequence, been more influenced by these emotionally positive stimuli while solving arithmetic problems. Such a possibility may be tested in future research by collecting eye movements and by determining whether pupil dilation (a measure of cognitive effort and attention) collected during processing of emotionally positive images differ in our younger and older groups of children.

The next interesting developmental findings here concerns age-related changes in sequential modulations of effects of negative and positive emotions. First, effects of negative emotions on current trials were smaller after negative than after neutral trials in all age groups, except in 12-year-old children. In this age group, effects of negative emotions were smaller after negative than after neutral trials but the Emotion on previous trial x Emotion on current trial interaction failed to reach significance. This suggests that in all age groups, except 12-year-olds, children prepared themselves on negative trials to process the next trials that could potentially be a negative trial in order to be less impaired by negative emotions. Most likely, 12 year-olds also did this, but probably not systematically or efficiently enough for the sequential modulations of effects of negative emotions to be significant. Future studies should determine whether this is specific to our sample of 12-year-old children or is replicated.

The sequential modulations of effects of positive emotions were significant in only one of our two groups of older children, namely the older group of 14-year-old children. Like for negative emotions, it is likely that 14-year-old children prepared themselves for the incoming trial after solving a problem under positive emotions, so as to be more focused on the arithmetic task and be less distracted by an incoming positive trial. That 12-year-old children did not sequentially modulate effects of positive emotions may be the result of this age group starting to pay more attention to positive stimuli. Consistent with this possibility, deleterious effects of positive emotions on performance were the largest in this age group.

In summary, our findings showed that both positive and negative emotions impaired children’s arithmetic performance, that deleterious effects of negative emotions were larger than those of positive emotions, that effects of emotions on current trials are influenced by emotions on immediately preceding trials, and that effects of emotions as well as their trial-to-trial modulations changed with children’s age. Both emotion-related changes in arithmetic performance and age-related changes in these differences are most likely the result of increased arithmetic fluency, better emotion regulation, and more efficient executive control with age.

This study includes a number of limitations. They concern conditions of occurrence of effects of emotion on children’s arithmetic performance at the empirical level and mechanisms underlying these effects of emotions at the theoretical level. Empirically, future studies may examine whether effects of negative and positive emotions interact with parameters characterizing task, stimuli, or situations. These include for examples asking whether effects of emotions vary with different arithmetic operations (e.g., addition vs. multiplication), with proportions of different types of problems (true/false; easier/harder), with different types of positive (e.g., happiness, enjoyment) and negative (e.g., anger, sadness, anxiety) emotions distinguishing activating (e.g., enjoyment, anger) or deactivating (e.g., relaxation, boredom) emotions as suggested by the CVT and previous correlational findings [4,19–21,28,30] as well as with varying situational parameters (e.g., speed/accuracy pressures).

Future studies will determine whether the effects found here hold across different types of positive or negative emotions, and whether the effects of incidental emotions (i.e., emotions that are induced by a situation that is independent of the task to be performed or the information needed to perform it, such as anxiety experienced by someone with an anxious personality or fear experienced while watching a scary movie) found here interact with previously found effects of integral emotions (i.e., emotions triggered by the task and/or the stimuli/information that must be processed to perform the task, such as anxiety provoked by mathematics). Alternatively, future studies will determine whether different effects are found for different emotions despite similar valence and/or whether mechanisms responsible for effects of incidental and integral emotions are the same or different. Indeed, previous studies suggest that different emotions may exert different effects in arithmetic [30]. Thus, stronger effects were found for happiness and sadness on participants’ cognition than for anger or anxiety [74, for an overview], and different electrophysiological signatures have been reported [49] with lower *N*1 amplitudes in fear than in anger condition while adults were solving complex arithmetic problems.

Another limitation of the present study is that our conclusion of age-related changes in effects of emotions on arithmetic performance need to be tested in different mathematical cognition tasks. For example, we found age-related decrease in deleterious effects of negative emotions on arithmetic performance (and deleterious effects of positive emotions on older participants’ performance). Such conclusions may hold across different arithmetic problems or tasks. They may also be different in situations where, for example, older children and adolescents are asked to solve harder math or arithmetic problems than simple arithmetic problems. One possibility is that with harder tasks or problems to solve, older children and adolescents’ attention would be more strongly capture by emotions than they are while solving simple arithmetic problems. Such a possibility could be tested by matching arithmetic task difficulty across age groups, so that older participants would solve harder problems (e.g., asking them to find approximate estimates two-digit multiplication problems) or harder math tasks (e.g., calculate sums of the surface of two rectangles).

Theoretically, one of the most important limitations of the present study concerns whether effects of emotions on children’s arithmetic occur via only one type of mechanism (e.g., attentional capture) as assumed here or whether other mechanisms (e.g., metacognitive judgment, inhibitory control, working memory) may cause children to perform more poorly under emotions. Other mechanisms that previous research revealed crucial in arithmetic [75] include strategic variations. Emotions may lead children to use fewer strategies, execute a given strategy more poorly, and/or to select poorer strategies on each problem), as this was recently found in adults [45]. Similarly, age-related changes in effects of emotions may be associated with age-related changes in which strategy dimensions are influenced by emotions. For example, poorer performance under negative emotions may be associated with poorer strategy selection in one age group, and with poorer strategy execution in another age group (or with both strategy dimensions, but to different extents, in both age groups). By examining emotion-related differences in strategic variations while children of different age groups solve arithmetic problems, future studies could determine whether, like in adults, emotions influence arithmetic performance via strategic differences.

## Supporting information

S1 Table Bayesian Linear Mixed Model on the influence of emotions (neutral, negative, positive) on arithmetic performance (response times). Analyses on the whole sample (*n* = 149).
(PDF)

S2 Table Bayesian linear Mixed Model of emotions (neutral, negative, positive) on arithmetic performance (response times). Analyses on the 8 years old (*n* = 35).
(PDF)

S3 Table Bayesian linear Mixed Model of emotions (neutral, negative, positive) on arithmetic performance (response times). Analyses on the 10 years old (*n* = 40).
(PDF)

S4 Table Bayesian linear Mixed Model of emotions (neutral, negative, positive) on arithmetic performance (response times). Analyses on the 12 years old (*n* = 34).
(PDF)

S5 Table Bayesian linear Mixed Model of emotions (neutral, negative, positive) on arithmetic performance (response times). Analyses on the 14 years old (*n* = 40).
(PDF)

S6 Table Bayesian Linear Mixed Model of the influence on emotions (neutral, negative, positive) on arithmetic performance (accuracy). Analyses on the whole sample (*n* = 149).
(PDF)

S7 Table Bayesian linear Mixed Model of emotions (neutral, negative, positive) on arithmetic performance (accuracy). Analyses on the 8 years old (*n* = 35).
(PDF)

S8 Table Bayesian linear Mixed Model of emotions (neutral, negative, positive) on arithmetic performance (accuracy). Analyses on the 10 years old (*n* = 40).
(PDF)

S9 Table Bayesian linear Mixed Model of emotions (neutral, negative, positive) on arithmetic performance (accuracy). Analyses on the 12 years old (*n* = 34).
(PDF)

S10 Table Bayesian linear Mixed Model of emotions (neutral, negative, positive) on arithmetic performance (accuracy). Analyses on the 14 years old (*n* = 40).
(PDF)
