# Supplementary material for: How do positive and negative emotions influence children’s and adolescents’ arithmetic performance?
Source: PLoS One. 2025 Apr 17;20(4):e0309573. doi: 10.1371/journal.pone.0309573 (PMC12005566; doi:10.1371/journal.pone.0309573)
Supplement: S1 Table — Analyses on the whole sample (n = 149). (PDF) [file pone.0309573.s001.pdf]

S1 Table.

Bayesian Linear Mixed Model on the influence of emotions (neutral, negative, positive) on arithmetic performance (responses times). Analyses on the whole sample (n = 149)

	Estimated coefficient	*SE*	*95% CI*	*Rhat*	*Bulk_ESS*	*Tail_ESS*
Population-level-effects						
(Intercept)	**13300.06**	**956.59**	[11412.56; 15170.62]	1.00	4500	9264
Emotion	**683.22**	**224.11**	[242.31; 1120.04]	1.00	33855	30938
Age	**-716.66**	**81.91**	[-877.31; -555.62]	1.00	4325	9198
Veracity	**-685.55**	**148.04**	[-976.13; -394.94]	1.00	69969	30523
Age*Emotion	**-40.63**	**18.85**	[-77.26; -3.51]	1.00	34803	31551
Emotion*Veracity	**-571.19**	**187.89**	[-942.12; -204.30]	1.00	35737	32169
Age*Emotion*Veracity	**48.67**	**14.83**	[19.52; 77.86]	1.00	388130	32533
Group-level-effects						
Sd(Intercept)	**2040.11**	**124.57**	[1813.46; 2301.01]	1.00	5570	9710
Family Specific Parameters						
sigma	**3606.56**	**22.48**	[3652.54; 3651.02]	1.00	95196	30231
Population-level-effects						

(Intercept)	**13659.86**	**925.98**	[11841.03; 15464.31]	1.00	2376	4638
Emotion negative	**3818.85**	**572.83**	[2694.88; 4951.58]	1.00	12411	23104
Emotion positive	91.23	577.20	[-1033.54; 1228.53]	1.00	12320	22817
Age	**-750.08**	**79.55**	[-904.67; -593.62]	1.00	2307	5008
Veracity	**-1467.92**	**471.51**	[-2390.49; -549.73]	1.00	12690	23998
Age*Emotion negative	**-208.67**	**48.83**	[-305.11; -113.23]	1.00	12328	22869
Age*Emotion positive	9.26	49.32	[-87.59; 105.57]	1.00	12366	22825
Emotion negative*Veracity	**-2376.36**	**807.11**	[-3973.86; -805.03]	1.00	11959	21921
Emotion positive*Veracity	546.45	819.45	[-1062.23; 2155.26]	1.00	12276	22577
Age*Emotion neutral *Veracity	**81.07**	**40.22**	[2.73; 159.85]	1.00	12698	24806
Age*Emotion negative *Veracity	**217.98**	**56.06**	[108.43; 328.46]	1.00	18518	28762
Age*Emotion positive *Veracity	44.59	57.20	[-68.02; 157.82]	1.00	19731	30732
Group-level-effects						
Sd(Intercept)	**2041.86**	**126.22**	[1812.93; 2309.52]	1.00	3632	8048
Family Specific Parameters						
sigma	**3606.56**	**22.48**	[3652.54; 3651.02]	1.00	95196	30231

Note. Gaussian processing including No-U-Turn (Hoffman & Gelman, 2014); significant effects are highlighted in bold letters *observations* = 12895*;* Group-levels = 149; *Rhat* = potential scale reduction factor on split chains (at converge, *Rhat* = 1); *Bulk_ESS* = bulk effective sample size; *Tail_ESS* = tail effective sample size; *SE* = Standard Error; *CI* = confidence intervall; Veracity is coded 0 = false problems and 1 = true problem.
