# Supplementary material for: How do positive and negative emotions influence children’s and adolescents’ arithmetic performance?
Source: PLoS One. 2025 Apr 17;20(4):e0309573. doi: 10.1371/journal.pone.0309573 (PMC12005566; doi:10.1371/journal.pone.0309573)
Supplement: S2 Table — Analyses on the 8 years old (n = 35). (PDF) [file pone.0309573.s002.pdf]

S2 Table.

Bayesian linear Mixed Model of emotions (neutral, negative, positive) on arithmetic performance (response times). Analyses on the 8 years old (n = 35)

	Estimated coefficient	*SE*	*95% CI*	*Rhat*	*Bulk_ESS*	*Tail_ESS*
Population-level-effects						
(Intercept)	**7348.43**	**566.18**	[6239.27; 8462.11]	1.00	4871	10459
Emotion	**450.14**	**131.57**	[190.25; 708.50]	1.00	38953	37488
Emotion*Veracity	**-349.79**	**103.96**	[-552.27; -146.17]	1.00	36915	36622
Group-level-effects						
Sd(Intercept)	3060.45	399.60	[2391.96; 3954.19]	1.00	6202	8708
Family Specific Parameters						
sigma	5309.34	71.82	[5169.93; 5451.44]	1.00	44442	38030
Population-level-effects						
(Intercept)	**7805.58**	**553.39**	[6713.45; 8888.99]	1.00	5457	10553
Emotion negative	**1398.66**	**350.91**	[713.96; 2083.48]	1.00	36080	39278
Emotion positive	293.39	347.10	[-382.34; 973.85]	1.00	37754	39559
Emotion neutral*Veracity	**-694.26**	284.17	[-1251.80; -136.87]	1.00	46627	39701
Emotion negative*Veracity	**-1133.81**	**404.69**	[-1927.51; -342.60]	1.00	44515	38942

Emotion positive*Veracity	-536.02	401.06	[-1317.00; 244.30]	1.00	45697	39125
Group-level-effects						
Sd(Intercept)	3052.70	396.50	[2390.84; 3942.14]	1.00	7462	10356
Family Specific Parameters						
sigma	5290.70	71.29	[5151.98; 5432.97]	1.00	62020	37210

Note. Gaussian processing including No-U-Turn (Hoffman & Gelman, 2014); significant effects are highlighted in bold letters*; observations* = 2772*;* Group-levels = 35; *Rhat* = potential scale reduction factor on split chains (at converge, *Rhat* = 1); *Bulk_ESS* = bulk effective sample size; *Tail_ESS* = tail effective sample size; *SE* = Standard Error; *CI* = confidence intervall; Veracity is coded 0 = false problems and 1 = true problems.
