# Supplementary material for: How do positive and negative emotions influence children’s and adolescents’ arithmetic performance?
Source: PLoS One. 2025 Apr 17;20(4):e0309573. doi: 10.1371/journal.pone.0309573 (PMC12005566; doi:10.1371/journal.pone.0309573)
Supplement: S3 Table — Analyses on the 10 years old (n = 40). (PDF) [file pone.0309573.s003.pdf]

S3 Table.

Bayesian linear Mixed Model of emotions (neutral, negative, positive) on arithmetic performance (response times). Analyses on the 10 years old (n = 40)

	Estimated coefficient	*SE*	*95% CI*	*Rhat*	*Bulk_ESS*	*Tail_ESS*
Population-level-effects						
(Intercept)	**5150.88**	**342.42**	[4472.03; 5819.92]	1.00	6732	13637
Emotion	**429.37**	**90.52**	[252.21; 607.69]	1.00	56906	39715
Emotion*Veracity	**-571.55**	**71.11**	[-709.41; -431.84]	1.00	58341	41918
Group-level-effects						
Sd(Intercept)	1935.85	233.50	[1539.99; 2454.05]	1.00	8191	10955
Family Specific Parameters						
sigma	4040.75	49.07	[3946.03; 4138.04]	1.00	65238	39563
Population-level-effects						
(Intercept)	**5385.70**	**331.04**	[4738.90; 6036.00]	1.00	4619	10017
Emotion negative	**2594.06**	**231.08**	[2142.28; 3048.84]	1.00	31243	35870
Emotion positive	-156.74	232.01	[-607.54; 298.57]	1.00	30776	36201
Emotion neutral*Veracity	**-775.67**	189.81	[-1148.77; -403.51]	1.00	38289	37176
Emotion negative*Veracity	**-2781.58**	**265.56**	[-3298.58; -2256.96]	1.00	38140	38845

Emotion positive*Veracity	-467.83	268.79	[-994.40; 54.89]	1.00	36787	37617
Group-level-effects						
Sd(Intercept)	1926.68	235.82	[1525.17; 2443.89]	1.00	6456	10550
Family Specific Parameters						
sigma	3949.29	47.77	[3857.16; 4044.93]	1.00	52521	40641

Note. Gaussian processing including No-U-Turn (Hoffman & Gelman, 2014); significant effects are highlighted in bold letters; *observations* = 2772; Group-levels = 35; *Rhat* = potential scale reduction factor on split chains (at converge, *Rhat* = 1); *Bulk_ESS* = bulk effective sample size; *Tail_ESS* = tail effective sample size; *SE* = Standard Error; *CI* = confidence intervall; Veracity is coded 0 = false problems and 1 = true problems.
