# Supplementary material for: How do positive and negative emotions influence children’s and adolescents’ arithmetic performance?
Source: PLoS One. 2025 Apr 17;20(4):e0309573. doi: 10.1371/journal.pone.0309573 (PMC12005566; doi:10.1371/journal.pone.0309573)
Supplement: S4 Table — Analyses on the 12 years old (n = 34). (PDF) [file pone.0309573.s004.pdf]

S4 Table.

Bayesian linear Mixed Model of emotions (neutral, negative, positive) on arithmetic performance (response times). Analyses on the 12 years old (n = 34)

	Estimated coefficient	*SE*	*95% CI*	*Rhat*	*Bulk_ESS*	*Tail_ESS*
Population-level-effects						
(Intercept)	**4008.92**	**313.15**	[3397.59; 4630.75]	1.00	5115	9245
Emotion	**494.32**	**69.77**	[357.50; 631.19]	1.00	41230	38623
Emotion*Veracity	**-283.97**	**54.50**	[-390.36; -177.11]	1.00	42209	38504
Group-level-effects						
Sd(Intercept)	1691.91	220.51	[1321.27; 2182.35]	1.00	6565	9360
Family Specific Parameters						
sigma	2893.92	37.74	[2820.95; 2968.71]	1.00	50153	35422
Population-level-effects						
(Intercept)	**4493.83**	**304.31**	[3899.98; 5094.71]	1.00	5183	9768
Emotion negative	**1105.79**	**180.59**	[748.25; 1458.10]	1.00	32356	36718
Emotion positive	**577.34**	**182.44**	[220.96; 936.96]	1.00	29384	34444
Emotion neutral*Veracity	**-497.10**	**149.16**	[-791.00; -206.00]	1.00	37389	38171
Emotion negative*Veracity	**-926.68**	**207.24**	[-1331.46; -517.93]	1.00	38911	37366

Emotion positive*Veracity	**-466.03**	**209.59**	[-875.47; 55.52]	1.00	35668	37615
Group-level-effects						
Sd(Intercept)	1693.03	224.24	[1318.26; 2193.04]	1.00	6445	9761
Family Specific Parameters						
sigma	2880.01	37.3	[2807.69; 2954.69]	1.00	48270	36015

Note. Gaussian processing including No-U-Turn (Hoffman & Gelman, 2014); significant effects are highlighted in bold letters*; observations* = 3009*;* Group-levels = 34; *Rhat* = potential scale reduction factor on split chains (at converge, *Rhat* = 1); *Bulk_ESS* = bulk effective sample size; *Tail_ESS* = tail effective sample size; *SE* = Standard Error; *CI* = confidence intervall; Veracity is coded 0 = false problems and 1 = true problems.
