# Supplementary material for: How do positive and negative emotions influence children’s and adolescents’ arithmetic performance?
Source: PLoS One. 2025 Apr 17;20(4):e0309573. doi: 10.1371/journal.pone.0309573 (PMC12005566; doi:10.1371/journal.pone.0309573)
Supplement: S5 Table — Analyses on the 14 years old (n = 40). (PDF) [file pone.0309573.s005.pdf]

S5 Table.

Bayesian linear Mixed Model of emotions (neutral, negative, positive) on arithmetic performance (response times). Analyses on the 14 years old (n = 40)

	Estimated coefficient	*SE*	*95% CI*	*Rhat*	*Bulk_ESS*	*Tail_ESS*
Population-level-effects						
(Intercept)	**2789.44**	**156.98**	[2479.99; 3099.67]	1.00	3653	8710
Emotion	**176.65**	**32.46**	[112.99; 240.36]	1.00	54261	41481
Emotion*Veracity	**-130.73**	**25.43**	[-180.43; -80.59]	1.00	53904	41168
Group-level-effects						
Sd(Intercept)	916.43	109.48	[732.24; 1157.94]	1.00	6765	9586
Family Specific Parameters						
sigma	1496.12	17.53	[1462.16; 1530.66]	1.00	68553	38103
Population-level-effects						
(Intercept)	**2943.45**	**150.69**	[2647.37; 3239.75]	1.00	3525	7729
Emotion negative	**566.08**	**84.84**	[398.42; 731.92]	1.00	33139	37549
Emotion positive	146.48	84.70	[-19.17; 314.07]	1.00	32770	36137
Emotion neutral*Veracity	**-224.15**	69.49	[-360.77; -86.89]	1.00	43021	41661
Emotion negative*Veracity	**-450.17**	**97.35**	[-641.02; -259.34]	1.00	42505	40408

Emotion positive*Veracity	**-207.59**	**98.28**	[-400.39; -14.35]	1.00	41789	40304
Group-level-effects						
Sd(Intercept)	915.75	110.40	[730.53; 1162.58]	1.00	5820	10470
Family Specific Parameters						
sigma	1485.94	17.45	[1452.27; 1520.75]	1.00	62419	40921

Note. Gaussian processing including No-U-Turn (Hoffman & Gelman, 2014); significant effects are highlighted in bold letters; *observations* = 3662; Group-levels = 40; *Rhat* = potential scale reduction factor on split chains (at converge, *Rhat* = 1); *Bulk_ESS* = bulk effective sample size; *Tail_ESS* = tail effective sample size; *SE* = Standard Error; *CI* = confidence intervall; Veracity is coded 0 = false problems and 1 = true problems.
