# Supplementary material for: How do positive and negative emotions influence children’s and adolescents’ arithmetic performance?
Source: PLoS One. 2025 Apr 17;20(4):e0309573. doi: 10.1371/journal.pone.0309573 (PMC12005566; doi:10.1371/journal.pone.0309573)
Supplement: S6 Table — Analyses on the whole sample (n = 149). (PDF) [file pone.0309573.s006.pdf]

S6 Table.

Bayesian Linear Mixed Model of the influence on emotions (neutral, negative, positive) on arithmetic performance (accuracy). Analyses on the whole sample (n = 149)

	Estimated coefficient	*SE*	*95% CI*	*Rhat*	*Bulk_ESS*	*Tail_ESS*
Population-level-effects						
(Intercept)	**.67**	**.06**	[.55; .79]	1.00	5956	12697
Emotion	.00	.02	[-.03; .03]	1.00	77262	42316
Age	**.02**	**.01**	[.01; .03]	1.00	5889	12212
Veracity	**.03**	**.01**	[.01; .05]	1.00	99533	42328
Age*Emotion	.00	.00	[-.00; .00]	1.00	82379	41808
Emotion*Veracity	-.02	.01	[-.05; .00]	1.00	77952	46104
Age*Emotion*Veracity	.00	.00	[-.00; .00]	1.00	88197	46833
Group-level-effects						
Sd(Intercept)	.13	.01	[.11; .14]	1.00	6378	11401
Family Specific Parameters						
sigma	.27	.00	[.26; .27]	1.00	75796	40524
Population-level-effects						

(Intercept)	.65	.06	[.54; .77]	1.00	6006	12903
Emotion negative	.02	.04	[-.06; .10]	1.00	34214	38755
Emotion positive	.06	.04	[-.02; .13]	1.00	33560	38659
Age	**.02**	**.01**	[.01; .03]	1.00	5922	12536
Veracity	**.07**	**.03**	[.01; .14]	1.00	36113	38925
Age*Emotion negative	-.00	.00	[-.01; .01]	1.00	34436	39576
Age*Emotion positive	-.00	.00	[-.01; .00]	1.00	33654	39290
Emotion negative*Veracity	**-.15**	**.06**	[-.26; -.03]	1.00	32259	38078
Emotion positive*Veracity	**-.12**	**.06**	[-.23; -.01]	1.00	32861	37767
Age*Emotion neutral *Veracity	-.01	.00	[-.01; .00]	1.00	36096	39256
Age*Emotion negative *Veracity	.01	.00	[-.00; .01]	1.00	52506	43231
Age*Emotion positive *Veracity	.00	.00	[-.00; .01]	1.00	51410	43344
Group-level-effects						
Sd(Intercept)	.13	.01	[.11; .14]	1.00	8633	10301
Family Specific Parameters						
sigma	.27	.00	[.26; .27]	1.00	103451	39774

Note. Gaussian processing including No-U-Turn (Hoffman & Gelman, 2014); significant effects are highlighted in bold letters *observations* = 14290*;* Group-levels = 149; *Rhat* = potential scale reduction factor on split chains (at converge, *Rhat* = 1); *Bulk_ESS* = bulk effective sample size; *Tail_ESS* = tail effective sample size; *SE* = Standard Error; *CI* = confidence intervall; Veracity is coded 0 = false problems and 1 = true problems.
