# Supplementary material for: How do positive and negative emotions influence children’s and adolescents’ arithmetic performance?
Source: PLoS One. 2025 Apr 17;20(4):e0309573. doi: 10.1371/journal.pone.0309573 (PMC12005566; doi:10.1371/journal.pone.0309573)
Supplement: S7 Table — Analyses on the 8 years old (n = 35). (PDF) [file pone.0309573.s007.pdf]

S7 Table.

Bayesian linear Mixed Model of emotions (neutral, negative, positive) on arithmetic performance (accuracy). Analyses on the 8 years old (n = 35)

	Estimated coefficient	*SE*	*95% CI*	*Rhat*	*Bulk_ESS*	*Tail_ESS*
Population-level-effects						
(Intercept)	**.83**	**.03**	[.76; .89]	1.00	5031	9136
Emotion	.00	.01	[-.01; .02]	1.00	49536	39250
Emotion*Veracity	-.00	.01	[-.02; .01]	1.00	52209	40570
Group-level-effects						
Sd(Intercept)	.19	.02	[.15; .24]	1.00	6361	8050
Family Specific Parameters						
sigma	.34	.00	[.33; .34]	1.00	51451	38363
Population-level-effects						
(Intercept)	**.81**	**.03**	[.74; .87]	1.00	4634	8325
Emotion negative	.02	.02	[-.02; .06]	1.00	30027	35408
Emotion positive	**.04**	**.02**	[.00; .08]	1.00	30229	35389
Emotion neutral*Veracity	**.04**	**.02**	[.01; .07]	1.00	36609	39288
Emotion negative*Veracity	-.02	.02	[-.06; .03]	1.00	36878	36231

Emotion positive*Veracity	-.04	.02	[-.08; .01]	1.00	37589	37536
Group-level-effects						
Sd(Intercept)	.19	.02	[.15; .24]	1.00	5859	9380
Family Specific Parameters						
sigma	.34	.00	[.33; .34]	1.00	54031	38729

Note. Gaussian processing including No-U-Turn (Hoffman & Gelman, 2014); significant effects are highlighted in bold letters; *observations* = 3360; Group-levels = 35; *Rhat* = potential scale reduction factor on split chains (at converge, *Rhat* = 1); *Bulk_ESS* = bulk effective sample size; *Tail_ESS* = tail effective sample size; *SE* = Standard Error; *CI* = confidence intervall; Veracity is coded 0 = false problems and 1 = true problems.
