# Supplementary material for: How do positive and negative emotions influence children’s and adolescents’ arithmetic performance?
Source: PLoS One. 2025 Apr 17;20(4):e0309573. doi: 10.1371/journal.pone.0309573 (PMC12005566; doi:10.1371/journal.pone.0309573)
Supplement: S8 Table — Analyses on the 10 years old (n = 40). (PDF) [file pone.0309573.s008.pdf]

S8 Table.

Bayesian linear Mixed Model of emotions (neutral, negative, positive) on arithmetic performance (accuracy). Analyses on the 10 years old (n = 40)

	Estimated coefficient	*SE*	*95% CI*	*Rhat*	*Bulk_ESS*	*Tail_ESS*
Population-level-effects						
(Intercept)	**.90**	**.02**	[.85; .94]	1.00	5413	10228
Emotion	.00	.01	[-.01; .01]	1.00	65658	40001
Emotion*Veracity	-.00	.00	[-.01; .01]	1.00	67328	41445
Group-level-effects						
Sd(Intercept)	.13	.02	[.11; .17]	1.00	8883	12856
Family Specific Parameters						
sigma	.27	.00	[.27; .28]	1.00	63969	38648
Population-level-effects						
(Intercept)	**.89**	**.02**	[.85; .94]	1.00	4496	9833
Emotion negative	.02	.02	[-.01; .05]	1.00	36800	38512
Emotion positive	.01	.02	[-.02; .04]	1.00	36223	38765
Emotion neutral*Veracity	.01	.01	[-.02; .03]	1.00	43689	40758
Emotion negative*Veracity	-.01	.02	[-.05; .02]	1.00	46370	41152

Emotion positive*Veracity	-.00	.02	[-.04; .03]	1.00	43762	40123
Group-level-effects						
Sd(Intercept)	.13	.02	[.11; .17]	1.00	9300	14502
Family Specific Parameters						
sigma	.27	.00	[.27; .28]	1.00	57084	38638

Note. Gaussian processing including No-U-Turn (Hoffman & Gelman, 2014); significant effects are highlighted in bold letters*; observations* = 3840*;* Group-levels = 40; *Rhat* = potential scale reduction factor on split chains (at converge, *Rhat* = 1); *Bulk_ESS* = bulk effective sample size; *Tail_ESS* = tail effective sample size; *SE* = Standard Error; *CI* = confidence intervall; Veracity is coded 0 = false problems and 1 = true problems.
