# Supplementary material for: How do positive and negative emotions influence children’s and adolescents’ arithmetic performance?
Source: PLoS One. 2025 Apr 17;20(4):e0309573. doi: 10.1371/journal.pone.0309573 (PMC12005566; doi:10.1371/journal.pone.0309573)
Supplement: S9 Table — Analyses on the 12 years old (n = 34). (PDF) [file pone.0309573.s009.pdf]

S9 Table.

Bayesian linear Mixed Model of emotions (neutral, negative, positive) on arithmetic performance (accuracy). Analyses on the 12 years old (n = 34)

	Estimated coefficient	*SE*	*95% CI*	*Rhat*	*Bulk_ESS*	*Tail_ESS*
Population-level-effects						
(Intercept)	**.93**	**.02**	[.88; .97]	1.00	5544	10247
Emotion	-.00	.01	[-.01; .01]	1.00	67579	40967
Emotion*Veracity	**.01**	**.00**	[.00; .02]	1.00	74855	41822
Group-level-effects						
Sd(Intercept)	.12	.02	[.09; .16]	1.00	10980	13756
Family Specific Parameters						
sigma	.24	.00	[.23; .24]	1.00	61728	39392
Population-level-effects						
(Intercept)	**.92**	**.02**	[.88; .97]	1.00	4619	9965
Emotion negative	-.00	.01	[-.03; .02]	1.00	38303	39624
Emotion positive	-.01	.01	[-.04; .02]	1.00	38799	39793
Emotion neutral*Veracity	.00	.01	[-.02; .02]	1.00	47924	41589
Emotion negative*Veracity	.03	.02	[-.01; .06]	1.00	45637	39510

Emotion positive*Veracity	.02	.02	[-.01; .06]	1.00	44359	39215
Group-level-effects						
Sd(Intercept)	.12	.02	[.09; .16]	1.00	9815	13956
Family Specific Parameters						
sigma	.24	.00	[.23; .24]	1.00	59301	38360

Note. Gaussian processing including No-U-Turn (Hoffman & Gelman, 2014); significant effects are highlighted in bold letters*; observations* = 3250*;* Group-levels = *3*4; *Rhat* = potential scale reduction factor on split chains (at converge, *Rhat* = 1); *Bulk_ESS* = bulk effective sample size; *Tail_ESS* = tail effective sample size; *SE* = Standard Error; *CI* = confidence intervall; Veracity is coded 0 = false problems and 1 = true problems.
