# Supplementary material for: How do positive and negative emotions influence children’s and adolescents’ arithmetic performance?
Source: PLoS One. 2025 Apr 17;20(4):e0309573. doi: 10.1371/journal.pone.0309573 (PMC12005566; doi:10.1371/journal.pone.0309573)
Supplement: S10 Table — Analyses on the 14 years old (n = 40). (PDF) [file pone.0309573.s010.pdf]

S10 Table.

Bayesian linear Mixed Model of emotions (neutral, negative, positive) on arithmetic performance (accuracy). Analyses on the 14 years old (n = 40)

	Estimated coefficient	*SE*	*95% CI*	*Rhat*	*Bulk_ESS*	*Tail_ESS*
Population-level-effects						
(Intercept)	**.95**	**.01**	[.92; .97]	1.00	18845	30817
Emotion	.01	.00	[-.00; .02]	1.00	101961	44166
Emotion*Veracity	-.00	.00	[-.01; .00]	1.00	105377	46346
Group-level-effects						
Sd(Intercept)	.05	.01	[.03; .06]	1.00	18172	28818
Family Specific Parameters						
sigma	.21	.00	[.20; .21]	1.00	91238	40953
Population-level-effects						
(Intercept)	**.94**	**.01**	[.92; .96]	1.00	14888	25405
Emotion negative	**.02**	**.01**	[.00; .05]	1.00	39928	41517
Emotion positive	**.03**	**.01**	[.00; .05]	1.00	36974	40444
Emotion neutral*Veracity	.01	.01	[-.01; .03]	1.00	51462	42520
Emotion negative*Veracity	.01	.01	[-.02; .04]	1.00	47211	42396

Emotion positive*Veracity	**-.03**	**.01**	[-.06; -.01]	1.00	45949	42539
Group-level-effects						
Sd(Intercept)	.05	.01	[.03; .06]	1.00	16814	26627
Family Specific Parameters						
sigma	.21	.00	[.20; .21]	1.00	72910	40700

Note. Gaussian processing including No-U-Turn (Hoffman & Gelman, 2014); significant effects are highlighted in bold letters; *observations* = 3840; Group-levels = 40; *Rhat* = potential scale reduction factor on split chains (at converge, *Rhat* = 1); *Bulk_ESS* = bulk effective sample size; *Tail_ESS* = tail effective sample size; *SE* = Standard Error; *CI* = confidence intervall; Veracity is coded 0 = false problems and 1 = true problems.
